# Speech therapy stimulation program for social skills of adolescents victims of mistreatment

**DOI:** 10.1192/j.eurpsy.2025.2180

**Published:** 2025-08-26

**Authors:** M. M. N. Matsumoto, A. A. Carneiro, D. R. Molini-Avejonas

**Affiliations:** 1 University of Sao Paulo, Sao Paulo, Brazil

## Abstract

**Introduction:**

The United Nations Children’s Fund defines mistreatment as any act that causes harm to the health, dignity or development of an individual. The aim of this study was to quantitatively analyze the effect of speech therapy stimulation on the development and quality of social skills in adolescents victims of childhood mistreatment. The hypothesis is that after the intervention program the subjects will show improvement in relation to the performance of these skills.

**Objectives:**

To quantitatively and qualitatively analyze the effect of speech therapy stimulation on the development and quality of social skills in adolescents victims of childhood mistreatment. The hypothesis is that after the intervention program the subjects will show improvement in relation to the performance of these skills.

**Methods:**

10 adolescents, between 12 and 16 years old, have participated in the research. The following inclusion criteria were used: 1) being between 7 and 17 years old; 2) having experienced mistreatment in childhood; 3) be treated in a highly complex health service. The intervention programs consisted of 12 follow-up sessions, carried out in person. The main focus was on stimulating communicative and pragmatic skills, also covering theory of mind skills, personal problem solving and paralinguistic skills. After the 12 sessions, patients underwent reevaluation and the results were tabulated and analyzed by the researcher.

**Results:**

Participants had considerable difficulties with social skills before speech therapy intervention, the initial difficulties was mainly in self-control and assertiveness skills. After the 12 intervention sessions, a significant improvement in the social skills was observed, especially in empathy and assertiveness.

**Conclusions:**

The participants progressed after the intervention in all areas evaluated, with more significant improvements in empathy and assertiveness. These results demonstrate the positive impact of speech-language therapy intervention for the stimulation of social communication and pragmatic skills.

It is important to emphasize that this population has many specificities, making it difficult to work with isolated variables. Therefore, more studies involving speech-language therapy, particularly speech-language therapy intervention, with adolescent victims of abuse are needed.

**Disclosure of Interest:**

None Declared

